# When vaccines reset tumors: SARS-CoV-2 mRNA shots create a transient checkpoint-sensitive state

**DOI:** 10.1038/s41392-025-02521-3

**Published:** 2025-12-31

**Authors:** Hao Chi, Michele Carbone, Youping Deng

**Affiliations:** 1 Department of Quantitative Health Sciences, John A. Burns School of Medicine, University of Hawaii at Manoa, Honolulu, HI USA; 2 Thoracic Oncology Program, University of Hawaii Cancer Center, Honolulu, HI USA

**Keywords:** Immunotherapy, Tumour immunology

In a recent study published in Nature, Grippin and colleagues showed that SARS-CoV-2 mRNA vaccines can transiently reset the tumor–immune interface, converting immunologically “cold” tumors into those that respond to PD-1/PD-L1 blockade. This work supports COVID-19 mRNA vaccines as systemic immune modulators that, when timed appropriately around immune checkpoint inhibitors (ICIs), can sensitize tumors and improve survival across diverse cancer types.^1^

**Fig. 1:**
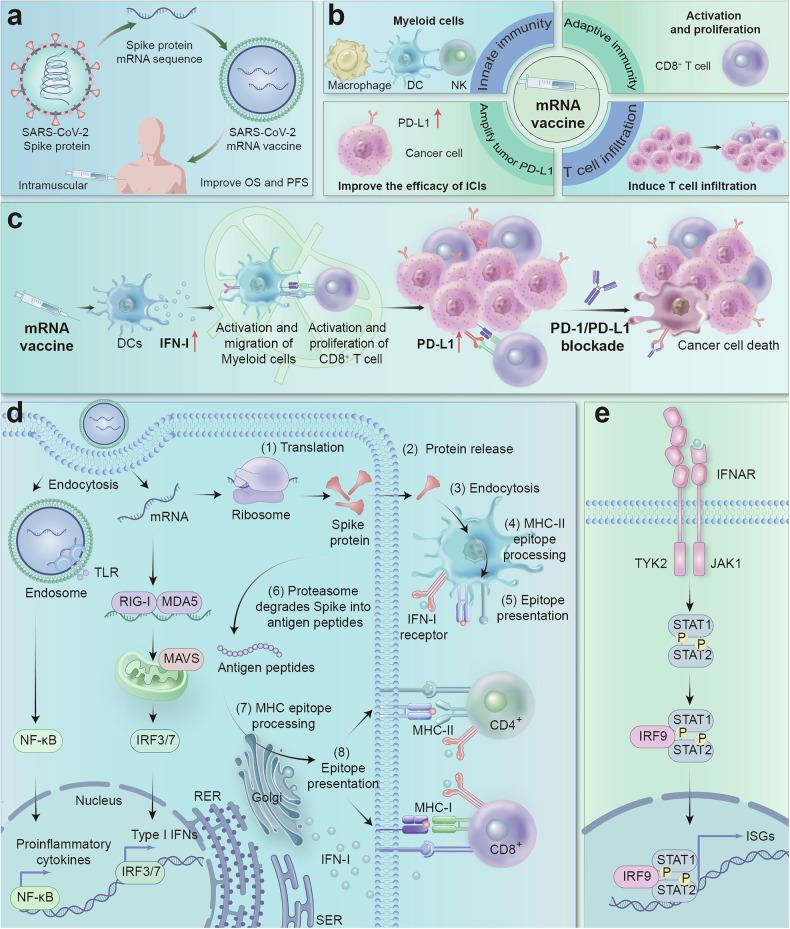
mRNA vaccines enhance tumor sensitivity to immune checkpoint inhibitors through immune modulation. **a** Design and efficacy of mRNA vaccines. **b** Activation of the immune system by mRNA vaccines. **c** mRNA vaccines enhance the response of “cold” tumors to immunotherapy. **d** Mechanism of the immune response induced by mRNA vaccines: targeted LNPs interact with specific receptors on the surface of antigen-presenting cells (APCs), and receptor activation may lead to the production of interferons and other cytokines/chemokines. After endocytosis, mRNAs in the endosome interact with membrane-bound Toll-like receptors (TLRs), triggering signaling pathways that activate the production of IFN-I and/or proinflammatory cytokines. The retained mRNA then undergoes endosomal escape and is released into the cytosol, where it is translated by ribosomes (1). The translated proteins can be secreted by the host cell (2). Secreted proteins are taken up by other APCs (3), degraded into peptides (4), and presented via MHC II molecules to activate CD4 + T cells (5). Alternatively, translated proteins are degraded into small peptides within the same cell (6), which are then transported to the endoplasmic reticulum and loaded onto MHC I or II molecules, which is a less common pathway (7). Finally, MHC-peptide complexes are presented on the surface of APCs and bind to the T-cell receptors (TCRs) of CD8+ and/or CD4 + T cells, triggering a specific immune response (8). Additionally, mRNAs interact with RIG-I and MDA5 receptors, triggering the production of IFN-I and proinflammatory cytokines and enhancing the immune response. **e** Activation of the IFN-I signaling pathway

In mouse tumors, spike mRNA lipid nanoparticles (RNA-LNPs) elicited IFNAR1-dependent innate activation that drove dendritic-cell priming and the expansion of cytotoxic CD8⁺ T-cell clones, accompanied by PD-L1 upregulation in tumor and myeloid compartments. Blocking type I interferon signaling abrogated both PD-L1 induction and synergy with anti-PD-1/PD-L1, pinpointing IFN-I as the mechanistic lynchpin and defining a short “interferon window” during which checkpoint blockade is maximally effective. To probe antigen specificity, the authors replaced spike with the human cytomegalovirus antigen pp65, which is overexpressed in human glioma but not in the B16F0 model, and observed comparable antitumour activity, indicating that the checkpoint-sensitive state is largely driven by antigen-agnostic sensing of the mRNA–LNP platform rather than spike-specific priming. They further modified the mRNA backbone, showing that replacing N1-methyl-pseudouridine with uridine in pp65 mRNA strengthened synergy with ICI, whereas the same change in spike mRNA conferred only modest numerical benefit—evidence that RNA chemistry and structure, independent of the encoded antigen, can tune the magnitude of IFN-I–mediated tumor sensitization.^1^ Together, these mouse data implicate platform-driven innate immunity, rather than spikes alone, as the primary driver of the transient checkpoint-sensitive state illustrated in Fig. 1.

Translational readouts aligned with these preclinical observations. In healthy volunteers, mRNA vaccination generated a brief IFN-α peak and broad innate/adaptive activation, including PD-L1 increases on circulating CD11b⁺ myeloid cells and CD11c⁺ dendritic cells that normalized by day 7. Pathology cohorts presented higher tumor PD-L1 tumor proportion scores when biopsies were collected <100 days post-vaccination than when they were collected in unvaccinated or ≥100-day windows. In ICI-treated patients, vaccination within 100 days of ICI initiation improved overall survival—the median OS increased from 20.6 to 37.3 months (adjusted HR ≈ 0.51) in a large NSCLC cohort; a tissue-agnostic analysis of ICI-treated patients also revealed benefits (HR ≈ 0.73). Notably, influenza or pneumococcal vaccines did not reproduce these survival gains, underscoring an mRNA platform-specific innate imprint; however, current clinical datasets cannot fully disentangle spike-specific recall responses from this generalized innate reprogramming, so future studies using nonspike mRNA platforms in patients will be informative.^1^

For a decade, interferon biology has framed ICI responsiveness: tumor-intrinsic IFN/IFN-γ signatures track with benefit, whereas defects in antigen presentation and JAK–STAT signaling herald resistance; however, chronic interferon programs can reinforce adaptive resistance. The new study operationalizes this continuum—the use of an acute IFN-I pulse to open the gate and then the application of PD-1/PD-L1 blockade to sustain T-cell function—so that antitumor immunity can be clocked from the periphery and then harnessed intratumorally.^2^ This concept resonates with prior work showing that IFN-γ–related expression profiles predict the PD-1 response and that a conserved IFN-γ transcriptomic program is beneficial for therapy, yet prolonged interferon exposure engrains inflammatory “memory” states linked to acquired resistance.^3^ The present paper clarifies the sweet spot: acute interferon to open the gate, followed by checkpoint blockade to sustain T-cell function while avoiding the liabilities of chronic IFN signaling.

A striking practical implication is that population-scale vaccination and precision oncology may intersect. If approved mRNA vaccines can function as on-demand innate adjuvants, then sequencing and dose (the paper notes lower innate activation with BNT162b2 than with mRNA-1273 at the studied doses) become controllable variables for designing “prime-and-unleash” schedules around ICI starts or restarts. Prospective trials should (i) define the duration of the interferon window; (ii) test varying mRNA doses and formulations; and (iii) prespecify biomarkers—plasma IFN-α peaks, transient myeloid PD-L1 induction, and short-term increases in the tumor PD-L1 TPS—to trigger ICI administration. Negative controls (for example, inactivated or protein vaccines) are essential to confirm the platform specificity suggested by the retrospective data.

In parallel, clinical protocols must respect the duality of interferon use. The same axis that “opens” tumors can, if sustained, drive T-cell dysfunction and stable resistance programs. Thus, window-of-opportunity designs with dense pharmacodynamic sampling, strict temporal coordination, and predefined stop rules for repeated boosting are warranted.

Finally, this study dovetails with our earlier perspective that viral immune stimulation can recalibrate tumor–host interactions.^4^ Here, a nontumor antigen vaccine acts as a systems-level immune modulator, not merely as an antigen source, reinforcing the idea that manipulating antiviral pathways can augment antitumor immunity across histologies.^4^
